# Thermal stability assessment of calcium monosulfoaluminate 12-hydrate by applying the in-situ X-ray diffraction method at 25–1250 °C

**DOI:** 10.1038/s41598-023-30919-y

**Published:** 2023-03-07

**Authors:** Dovile Rubinaite, Tadas Dambrauskas, Kestutis Baltakys, Harald Hilbig, Raimundas Siauciunas

**Affiliations:** 1 Department of Silicate Technology, Kaunas University of Technology, Radvilenu pl 19, LT – 50254 Kaunas, Lithuania; 2 Professorship for Mineral Construction Materials, TUM School of Engineering and Design, Technical University of Munich, Franz-Langinger-Str. 10, 81245 Munich, Germany

**Keywords:** Ceramics, Composites

## Abstract

In this study, the stability of synthetic calcium monosulfoaluminate and the reaction mechanism of its conversion into ye`elimite during the thermal treatment were examined. The monosulfoaluminate was produced referring to ye`elimite stoichiometry by applying the mechanochemical treatment (dry grinding at 900 rpm with 3 on–off cycles of 10 min) followed by the hydrothermal synthesis (for 8 h at 110 °C). The data indicated that the prepared sample consists of Ms12 (~ 54.8%), CaCO_3_ (~ 1.9%), Ms10.5/Hc (~ 0.7%) and amorphous content (~ 42.6%). Meanwhile, the thermal stability assessment by in-situ XRD analysis reveals that the dehydration of monosulfoaluminate interlayer water proceeds at 25–370 °C, where four different hydration states of monosulfoaluminate are identified. Additionally, the results suggest that the removal of water molecules from the main (octahedral) layers begins at ~ 200 °C. Finally, at 700–1250 °C, the solid-state reactions between CŜ, CA and CaO are observed, generating the formation of ye`elimite.

## Introduction

It is recognised that in the CaO-Al_2_O_3_-CaSO_4_-H_2_O system, two main existing phases, AFt (ettringite) and AFm (monosulfoaluminate), can be distinguished. These phases mainly form during the hydration of Portland cement and calcium sulfoaluminate/aluminate-containing binders^1–3^. Both ettringite (C_6_AŜ_3_H_32_) and monosulfoaluminate (C_4_AŜH_12_) precipitate from lime, aluminium and calcium sulphate solutions. In the case of ettringite formation, a sufficient amount of additional calcium sulphate is needed^4,5^. Meanwhile, the deficiency of calcium sulphates results in the formation of monosulfoaluminate (C_4_AŜH_12_). In addition, ettringite can convert to monosulfoaluminate when sulphate is consumed and vice versa^1,6^.

Regarding the stability of these phases, it is essential to comprehend their behaviour in given conditions for concrete sustainability and durability. To date, the stability of these phases has been the subject of numerous studies that have raised many discussions and controversies. It is acknowledged that the stability of these phases highly depends on environmental conditions such as temperature, pressure, and relative humidity^7,8^. However, regarding the thermal stability of monosulfoaluminate, it is not as extensively examined as ettringite^9–12^. The main reason for this is the complexity of synthesising the pure hydration states of monosulfoaluminate and difficulties in experimental characterisation caused by their poor crystallinity. Thus, there are still some serious questions about the mechanism and reaction kinetics of monosulfoaluminate degradation that have not been thoroughly answered.

Fundamentally AFm phases are hydrated tetracalcium aluminate-ferrite minerals belonging to the layered double hydroxide family (LDH-type)^13^. The structure consists of positively charged main layers [Ca_2_(Al,Fe)(OH)_12_]^+^, which are compensated by negatively charged interlayers [X·nH_2_O]. Particularly this paper focuses on the aluminium AFm phase containing sulfate (SO_4_^2−^) anions, identified as calcium monosulfoaluminate 12-hydrate (monosulfate or monosulfoaluminate). As mentioned before, it is a prevailing compound in cement hydrate systems and has the naturally occurring structure analogue – mineral Kuzelite (C_4_AŜH_12_), as was characterised by Allmann^14^. The essential feature of the monosulfoaluminate structure is that the water content of interlayers may alter from 2 to 10 molecules, depending on the exposure to temperature and relative humidity^15^. Therefore, different hydration states of monosulfoaluminate are commonly denoted by an abbreviation followed by an index indicating the water content in the interlayer. This denotation has also been used in this work; for instance, calcium monosulfoaluminate 12-hydrate is denoted as Ms12.

Despite the difficulties of producing monosulfoaluminate, it can be synthesised by applying various methods. The first synthesis of monosulfoaluminate was reported by H. J. Kuzel in 1965^16^. The lab-scale production process was performed using a stoichiometric mixture of tricalcium aluminate (C_3_A) and gypsum (CaSO_4_·2H_2_O), applying hydrothermal treatment at 150 °C for 4 days. In comparison, scientists have also synthesised monosulfoaluminate using the thermal decomposition (commonly at ~ 114 °C) method on ettringite^17^. However, the drawbacks of this process include the complexity of synthesis and the occurrence of calcium sulphate in the final products. Nowadays, C_3_A and gypsum (suspended in a 1:1 molar mixture) are commonly used as precursors for the synthesis when the prepared suspension is stirred periodically for two weeks at 80 °C^15,18^.

The phenomenon of multiple transformations accompanied by mass change during the thermal decomposition of calcium monosulfoaluminate has already been successfully observed in multiply works. The decomposition/dehydration process of monosulfoaluminate has been extensively studied by H. Pöllmann (1984)^19^. He has reported that the loss of the six interlayer water molecules proceeds between 43 and 200 °C. Meanwhile, the six remaining water molecules from the main layers are lost between 200 and 300 °C. Other authors have suggested that the crystalline water from the octahedral layer is lost in slightly higher temperatures (250–300 °C)^20–22^. Accordingly, the remaining mass loss beyond this temperature is associated with the dehydration of the main layers. However, most of the previous studies dealing with the stability of monosulfoaluminate were mainly validated by thermal analysis (DSC/TG) and ex-situ XRD experimental results. These results do not demonstrate the precise mechanism and kinetics of degradation reactions of monosulfoaluminate, leaving not well-resolved questions about the decomposition of monosulfoaluminate.

High-temperature X-ray in-situ analysis is a powerful tool that allows real-time observations of phases transition and assists in revealing the reaction mechanism^23^. Additionally, this method enables the detection of intermediate phases in reactions or high-temperature phases that recrystallise to low-temperature phases during the cooling processes. The features of this method are essential for the correct study of the phase transitions of different hydration states of monosulfoaluminate since the ambient conditions significantly affect their stability. Only a few scientists have attempted to apply in-situ method measurements to investigate its stability. N. Meller has presented the hydrothermal decomposition of calcium monosulfoaluminate 14-hydrate by applying the in-situ synchrotron X-ray diffraction method^17^. Meanwhile, H. Pöllmann characterised the stability of kuzelite by in-situ X-ray investigation^24^. Nevertheless, none of the protocols describes detailed experimental conditions; one piece of information is always missing. In addition, there is a lack of elaboration on the obtained results, such as the beginning and end temperatures of the phases’ transition. Therefore, the answers to open-ended questions such as the stability of calcium monosulfoaluminate remain obscure.

Thus, synthetic calcium monosulfoaluminate dehydration sequences and the formation of crystalline phases mechanism at elevated temperatures are highlighted in this study. For the synthesis of calcium monosulfoaluminate, the mechanochemical-hydrothermal treatment approach was used. Meanwhile, the thermal stability was investigated by applying the in-situ XRD method and complemented with simultaneous thermal analysis. Finally, the results provide new insights into the mechanism behind the observed dehydration of calcium monosulfoaluminate.

## Materials and methods

### Raw materials

For the synthesis of calcium monosulfoaluminate, reagent-grade materials were used:

- Calcium carbonate (CaCO_3_, *Eksparas*, Lithuania), with purity ≥ 99.0 wt.% of CaCO_3_;
- Aluminium hydroxide (Al(OH)_3_, *Honeywell*, Germany) with purity ≥ 99.0 wt.% of Al(OH)_3_;
- Gypsum (CaSO_4_·2H_2_O, *Lach–Ner*, Poland), which consisted of 27.07 wt.% of Ca, 20.64 wt.% of S, and other substances (up to ~ 1% wt.%).

The composition of the initial mixture was prepared based on ye`elimite (Ca_4_Al_6_O_12_(SO_4_) stoichiometry. Calcium oxide (CaO) was obtained from calcium carbonate calcinated at 950 °C temperature for 1 h (the loss on ignition ~ 42.9%), where the quantity of free CaO was equal to ~ 98.2 wt.%. Meanwhile, the aluminium oxide (Al_2_O_3_) was prepared by dehydration of aluminium hydroxide at 475 °C temperature for 4 h (the loss on ignition ~ 34.2%). Both mentioned substances were sintered in an electric muffle furnace *SNOL 8.2/1100* (Umega group, AB, Lithuania).

### Synthesis process

**Figure 1:**
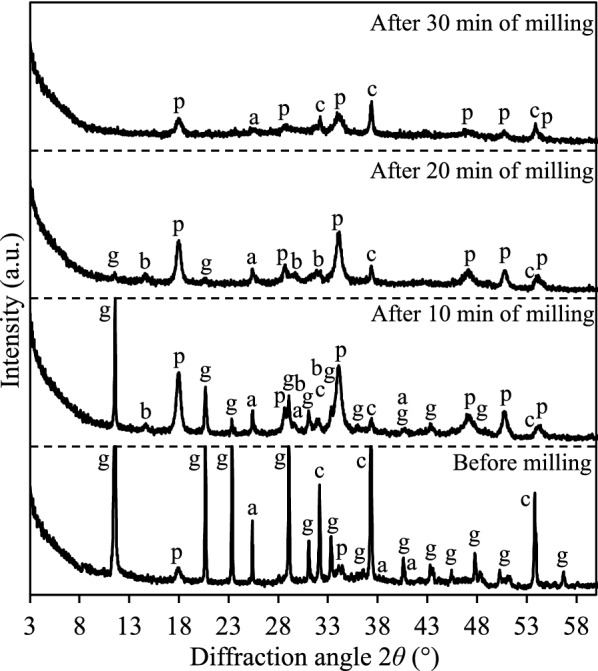
XRD patterns of the initial mixture at the different stages of mechanochemical treatment. The X-rays with a wavelength of 1.5406 Å (CuKα radiation) were used. Indexes: g – gypsum, p – portlandite, c – calcium oxide, a – anhydrite, b – bassanite.

Synthesis of calcium monosulfoaluminate was performed by applying the mechanochemical treatment and hydrothermal synthesis. In the first stage, the mechanical treatment of the initial mixture was performed using a high-energy vibrating cup mill *Pulverisette 9*. The grinding operated at 900 rpm with three on–off cycles of 10 min. It was observed that the mechanochemical treatment causes great structure and phase composition changes in the initial mixture. It was confirmed by XRD measurements performed before and after grinding for different durations (Fig. 1).

**Figure 2:**
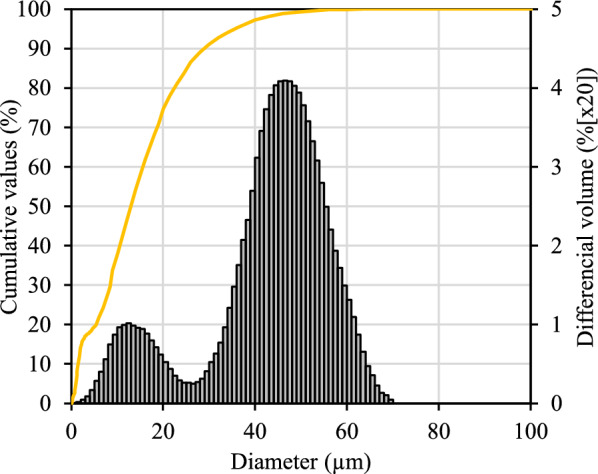
Particle size distribution of the ground mixture after 30 min.

The results show that within the first 10 min of milling, the mechanochemical treatment induces dehydration of gypsum, i.e., a significant decrease in the diffraction peaks characteristic of gypsum (CaSO_4_·2H_2_O, PDF No. 04-010-9409) is observed^25^. Additionally, due to partial water evaporation out of the gypsum structure, bassanite (CaSO_4_·0.5H_2_O, PDF No. 00-033-0310) is detected in the XRD patterns. At the same time, prepared calcium oxide (CaO, PDF No. 00-037-1497) reacts with water resulting in the formation of portlandite (Ca(OH)_2_, PDF No. 00-044-1481). Meanwhile, mechanochemical milling induces damage in the crystalline structure of anhydrite (present from the raw material) (CaSO_4_, PDF No. 00-037-1496), causing its reflections of diffraction peaks to reduce. This tendency is seen with further milling (up to 30 min) for almost all diffraction peaks of compounds of the initial mixture. It is evident that mechanochemical treatment induces the disintegration of the structure of the crystalline phases. The exception is observed only for calcium oxide, which reflections increase with increasing milling duration due to portlandite dehydration. It is worth mentioning that alumina exhibits an amorphous nature if it is produced by dehydration of Al(OH)_3_ below 700 °C^26,27^. Therefore, in this case, aluminium oxide is not seen in the XRD patterns. After the processing, the ground powder particle size distribution (present in Fig. 2) and density (3380 kg/m^3^) were measured.

In the second stage, the mechanochemical activated powder was treated in a hydrothermal environment. Firstly, the necessary amount (2 g) of powder was weighed, poured into 25 mL *PTFE* cells, and stirred with distilled water to reach a water/solid ratio equal to 10. The stirring was performed using a laboratory spatula for ~ 1 min. The hydrothermal synthesis was carried out in unstirred suspension autoclave *Parr instruments 4621* (Germany). The synthesis temperature (110 °C) was reached within 2 h, and the samples were stored for 8 h. An essential part of this process was the cooling stage—The suspension must be taken out not lower than 60 °C. It was observed that cooling down the suspension in the autoclave below 60 °C promotes ettringite formation. This observation coincides well with D. Damdot and F. P. Glasser estimations^28^, demonstrating that above 45 °C temperature, monosulfoaluminate is significantly more stable compared to ettringite. Meanwhile, recrystallisation of monosulfoaluminate to AFt, C_3_AH_6_ and gibbsite is possible at lower temperatures. After hydrothermal treatment, the suspension was decanted, rinsed with acetone, and filtered. For X-ray diffraction measurement, the wet sample was used directly after filtration. Meanwhile, the dry sample was prepared by drying at 50 ± 0.2 °C for 24 h in a dryer *SP-25 EASY* (Kambič, Slovenia). The obtained dry samples were sieved through a sieve with a mesh width of 80 µm to ensure particle distribution.

### Methods

The particle size distribution of the prepared initial mixture was analysed by °*ILAS LD 1090* LD grain-size analyser (*Cilas, Orleans*, France), and the density was by automatic gas pycnometer *Ultrapyc 1200e Quantachrome Instruments* (*Micromeritics*, USA).

The elemental compositions of materials were analysed by the X-ray fluorescence spectroscopy (XRF) analytical technique. The analysis was performed by a Bruker X-ray *S8 Tiger WD* (Bruker AXS GmbH, Karlsruhe, Germany) spectrometer equipped with the Rh tube (60 keV), the measurement atmosphere – He. The analysis was carried out on samples after passing through a 63 μm sieve and then compressed (20 MPa for 45 s) into 5 × 40 mm cylindrical tablets. The obtained data were analysed with *SPECTRAPlus QUANT EXPRESS* standardless software.

The X-ray diffraction analysis (XRD) was carried out to determine the crystallographic structure of materials. The measurements were performed at room temperature with the *D8 Advance* diffractometer (*Bruker AXS GmbH*, Karlsruhe, Germany) equipped with a CuKα X-ray tube operated at 40 kV and 45 mA. Diffraction patterns were recorded in the Bragg–Brentano geometry using a fast-counting detector *Bruker LynxEye* based on silicon strip technology. The measurement range was 2*θ* = 3–70° with steps of 0.020° 2*θ* with the time per step 0.2 s, corresponding to a total measurement duration of ~ 12 min per sample.

The quantitative content of the crystalline phases was refined by the Rietveld method as implemented in the *TOPAS 4.2 software* (*Bruker AXS GmbH, Karlsruhe, Germany).* The quantitative phase analysis (QPA) was carried out on samples after grinding by hand to pass a 40 µm sieve, ensuring an isotropic distribution of the crystals in the sample. For the quantitative determination of the amorphous phase, 20% of the internal standard (ZnO) was added to the sample. Additionally, the sample holder was rotated during the data recording to improve the particle statistics and obtain high-quality QPA data. The crystal structures used in the refinements were adopted from PDF 2022 database.

The thermal stability of monosulfoaluminate was investigated by applying the XRD in-situ method with the *MTC-HIGHTEMP* chamber (*Bruker AXS*, Karlsruhe, Germany). The samples for the measurement were prepared by mixing the powder (~ 0.2 g) with ethanol (96%) until the viscous consistency was reached. Afterwards, the thin suspension layer was placed on the heating strip. The experiments were performed in the temperature range of 25–1250 °C, with 50 °C/min, in an air atmosphere. The patterns were recorded after equilibration of 2 min at the desired temperature in the range 2*θ* = 8–38° with steps of ~ 0.02° 2*θ* and 0.2 s counting time per step. In total, 78 patterns were recorded within ~ 11.5 h.

Simultaneous thermal analysis (STA: differential scanning calorimetry – DSC and thermogravimetry – TG) measurements of synthesis products were carried out using a *LINSEIS STA PT 1000* (*Linseis Massgeraete GmbH, Selb*, Germany) thermal analyser with the type S thermocouple. The sample (10 ± 0.02 mg) was heated in the temperature range of 30–950 °C at a heating rate of 5 °C/min. The analysis was performed in the standard closed Pt-10 wt. % Rh crucibles under nitrogen flow, and the measurement accuracy reached ± 3 °C. The obtained results were analysed using the *Linseis Platinum Evaluation* software. Meanwhile, the additional DSC was performed with a Netzsch Polyma DSC 214 analyser (Netzsch, Germany) with the type E thermocouple. Measurement parameters: temperature heating rate – 5 °C/min, temperature range – 15–400 °C, the crucible – aluminium (Al) crucible with a lid, the atmosphere in the furnace – nitrogen, measurement accuracy – ± 3 °C. The data were assessed with NETZSCH Proteus software.

The Fourier transform infrared spectroscopy (FTIR) analysis was carried out using *the Perkin Elmer FT–IR Spectrum X system* (*PerkinElmer*, Waltham, MA, USA). The samples were analysed using the KBr pressed pellet technique (~ 1 mg of sample and ~ 200 mg of KBr), and spectra were recorded in the range of 4000–400 cm^−1^ with a spectral resolution of 1 cm^−1^.

The morphology of the samples was investigated by scanning electron microscopy (SEM) (JEOL JSM–7600F, Japan). The micrographs were obtained using an accelerating voltage of 10 kV under a high vacuum at a working distance of 5.0 mm and 4.9 mm.

The ^27^Al NMR spectroscopy was carried out with a *Bruker Avance 500* spectrometer (Bruker, Germany) with a magnetic field strength of 11.747 T (resonance frequency for ^27^Al: 130.308 MHz). The measurements were performed applying a single pulse MAS (magic angle spinning) technique. The samples were placed in a 4 mm zirconia rotor and spun at 12 kHz with a repetition time of 0.5 s, recording a total of 2000 scans. The ^27^Al chemical shifts were referenced relative to the Al(OH)_3_. The obtained spectra patterns were assessed using *WINNMR* software.

## Results and discussion

### Mineralogical composition and microstructure of the hydrothermal synthesis product

**Figure 3:**
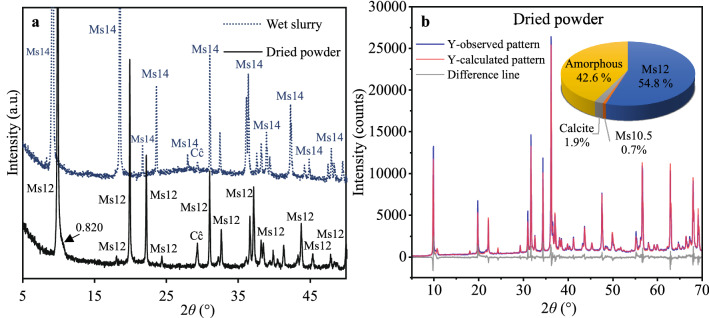
XRD patterns (**a**) and QPA results (**b**) of the samples after hydrothermal synthesis. The X-rays with a wavelength of 1.5406 Å (CuKα radiation) were used. Indexes: Ms –monosulfoaluminate (the number shows water content); Cĉ – CaCO_3_.

In the first stage of this research, the mineralogical composition and microstructure of the hydrothermal synthesis products were investigated. In order to evaluate the drying impact on crystalline phase assemblage, wet and dry samples were used for XRD diffraction analysis. As shown in the XRD patterns in Fig. 3a, calcium monosulfoaluminate 14-hydrate (Ms14) (Ca_4_Al_2_O_6_(SO_4_)·14H_2_O, PDF No. 00–042-0062) is observed as the main crystalline phase directly after the hydrothermal synthesis. Meanwhile, after the drying process (at 50 °C for 24 h), a lower hydration state is detected, corresponding to calcium sulfoaluminate 12-hydrate (Ms12) (Ca_2_(SO_4_)_0.5_(OH)_6_(H_2_O)_3_, PDF No. 04-013-03303). The results clearly show that Ms14 is a transition hydrate that loses two interlayer water molecules exposed to elevated temperature and dehydrates into Ms12. In addition, calcium carbonate (CaCO_3_, PDF No. 01-080-2793) and a small broad diffraction peak at ~ *d* – 0.820 nm are observed in the XRD patterns of dried samples. The detected broadening can be ascribed to the formation of calcium hemicarboaluminate (Hc) or calcium monosulfoaluminate 10.5-hydrate. The mentioned compounds could have been formed through Ms12 interaction with atmospheric CO_2_ or CaCO_3_ and/or partial Ms12 dehydration as Hc and Ms10.5, respectively^18^. However, the peak intensity is too low to identify the true nature of the formed phase. For further study, analysis methods were applied only to dried samples.

For the quantitative phase analysis, it was assumed that the diffraction peak at ~ *d* – 0.820 reflects the Ms10.5 structure. As the crystal structure patterns of the lower monosulfoaluminate hydration states (below Ms12) remain vague, the crystal structure of kuzelite was used as a starting model for Ms10.5 refinement. Aiming for adequate QPA data results, the space group and initial lattice parameters were adjusted considering the previous studies^15^. The phase composition results of the sample are presented in Fig. 3b.

**Figure 4:**
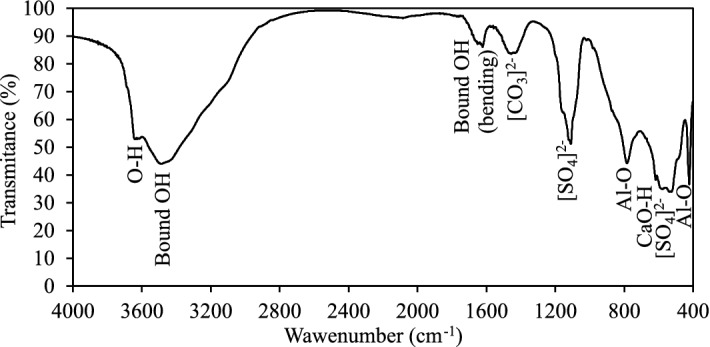
FTIR spectrum of the sample after hydrothermal synthesis.

The further characterisation of the sample after hydrothermal synthesis was done by FTIR spectroscopy which confirmed and supplemented the XRD analysis results. As shown in Fig. 4, two distinct regions are detected in the FTIR spectrum. The first, high-frequency region (4000–2500 cm^−1^) distinctly represents the OH stretching vibrations. The band at frequencies of 3635–3637 cm^−1^ indicate OH-stretching and confirms the presence of amorphous aluminium hydroxide (AH_3_). Furthermore, the broad absorption band, recorded between 3600 and 2900 cm^−1^, corresponds to the stretching vibrations of hydroxyl groups in the interlayer and the main layer of the monosulfoaluminate. Meanwhile, the second low-frequency region of the spectrum is seen below 2000 cm^−1^. In this region, the interpretation of the experimental data is more complex since many overlaps of the vibrational bands are observed. The detected absorption band at 1630 cm^−1^ corresponds to the bending vibration of the interlayer water molecules. Furthermore, the absorption band at 1455 cm^−1^ and 875 cm^−1^ shows asymmetric stretching vibration of CO_3_, indicating the presence of calcium carbonate. Towards the lower energies (below 1200 cm^−1^) are indicated absorptions of the SO_4_^2-^ (stretching at 1109 cm^−1^, bending at 423 cm^−1^), Al–O–H (bending at 785 cm^−1^ and 617 cm^−1^) and AlO_6_ vibration (at 588 cm^−1^, 522 cm^−1^). The interpretation of the FTIR spectrum bands agrees well with the previously published data^29–31^.

**Figure 5:**
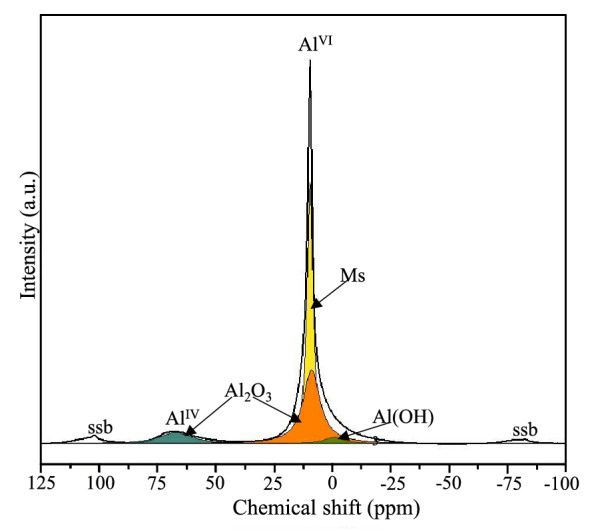
^27^Al NMR spectrum of the sample after hydrothermal synthesis.

The ^27^Al NMR analysis was used to examine the amorphous nature of the sample. The obtained data of the sample is presented in Fig. 5. The ^27^Al NMR spectrum of the sample shows two resonance ranges at the higher (from 80 to 45 ppm) and lower (from 30 to – 30 ppm) fields, reflecting resonances for fourfold and sixfold coordinated Al positions, respectively^32^. The dominant peak at ~ 9.4 ppm is a characteristic signal of AlO_6_ in monosulfoaluminate. Meanwhile, a broad shoulder at ~ 67 ppm corresponds to the presence of unreacted Al_2_O_3_, with the resonance of the sixfold coordinated aluminium part being hidden under the resonance of the monosulfoaluminate. According to XRD and ^27^Al NMR results, mainly amorphous content consists of unreacted Al_2_O_3_. In addition, the deconvolution demonstrates a small peak at 0–2 ppm, indicating the presence of a small amount of AH_3_ in the sample.

**Figure 6:**
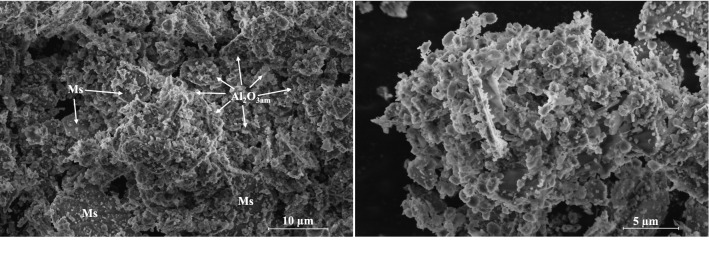
SEM micrographs of the sample after hydrothermal synthesis.

Further, the SEM analysis was used to identify the characteristic morphology of phases (Fig. 6). The images demonstrate that the sample contains particles of various sizes and morphologies. The most widely spread morphology is seen as a hexagonal plate as well as layered plates without defined borders, both corresponding to calcium monosulfoaluminate (size ~ 5–20 µm)^8^. Additionally, these structures are surrounded by brighter various size irregular-shaped agglomerates reflecting the morphology of amorphous Al_2_O_3_/Al(OH)_3_^27,32^. Due to the high quantity of amorphous Al_2_O_3_ and similar morphology, Al(OH)_3_ was not distinguished in SEM images.

### Thermal stability

**Figure 7:**
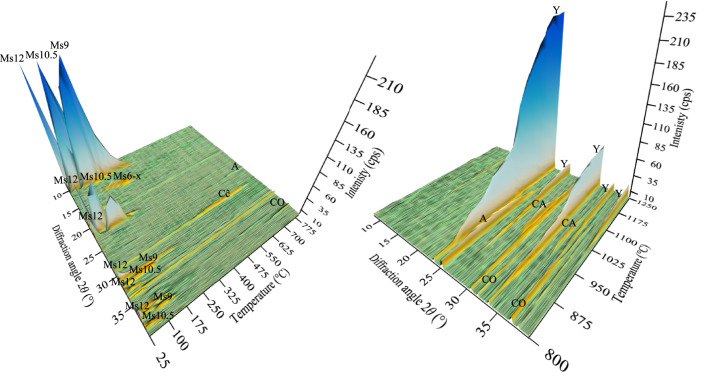
In situ XRD patterns of the synthesis products when the calcination temperature varied in a 25–1250 °C range. The X-rays with a wavelength of 1.5406 Å (CuKα radiation) were used. Indexes: Ms – monosulfoaluminate (the number shows water content); Cĉ – calcium carbonate; CO – calcium oxide; A – anhydrite; CA – calcium aluminate; Y – ye`elimite.

The structural changes associated with the thermal decomposition of the synthesis products were investigated by applying the in-situ X-ray diffraction method and simultaneous thermal analysis. It is worth noting that XRD patterns were recorded at the process temperature. Therefore, the thermal-induced shifts are visible for the positions of the peaks in the XRD patterns. Two main temperature intervals can be distinguished based on the results (Fig. 7). The first one (the phase decomposition interval) is seen in the temperature range of 25–800 °C, which is associated with dehydration and decarbonisation. Immediately after incrementing temperature, the intensity of peaks corresponding to Ms12 (main peak at *d* – 0.893 nm) begins to decrease, reflecting Ms12 dehydration. Meanwhile, the Ms12 completely dehydrates at ~ 90 °C. At the same time, the formation of Ms10.5 (*d* – 0.814 nm) at ~ 50 °C is observed, generated by the dehydration of Ms12. The increase in the intensity of Ms10.5 peaks is seen up to ~ 80 °C. Meanwhile, further temperature increase adversely affects the stability of Ms10.5, causing a gradual decrease in the intensity of peaks that are no longer visible at 220 °C. Similarly, the formation of a lower hydration state is observed at 90 °C, corresponding to Ms9 (*d* – 0.796 nm). The highest intensities of its peaks are seen at ~ 140 °C, while peaks remain visible even up to ~ 370 °C. These results indicate that the dehydration of Ms10.5 presumably begins at ~ 80 °C, causing the formation of Ms9. In the same way, the dehydration of Ms9 presumably begins at around ~ 140 °C, yielding the appearance of lower hydration states of monosulfoaluminate. As a result, a broad low-intensity diffraction peak at *d* – 0.617 nm is observed at 170–330 °C. Unfortunately, based on the PDF-4 database, the detected peak could not be determined. Nevertheless, this peak might indicate the transitional Ms6-x phase (x is water molecules, might be from 0 to 6), most likely it is Ms6, which may have an amorphous/low crystallinity nature. However, a high amount of formed compound and/or elevated ambient temperatures might have contributed to the peak detection. Additionally, the temperature (~ 200 °C) at which the peak intensity begins to decrease might show the beginning of the water elimination out of Ms main (octahedral) layers.

It is important to emphasise that only the main peak positions of the lower monosulfoaluminate states (Ms10.5 and Ms9) were presented in the previous studies^15,18^. Therefore, in this work, the unclear Ms positions were assigned based on the main phase peak thermal stability. For instance, the main Ms10.5 peak is detected at 50 °C and remains visible up to 220 °C. According to this tendency, appeared and maintained peaks at this temperature interval were assigned to this hydrate state. The identified thermal stability of monosulfoaluminate states and assigned peak positions are summarised in Table. 1.

**Table 1:** Thermal stability of different hydration states of calcium monosulfoaluminate states.

Phase	Stable in the temperature interval	Interplanar distance (this work), nm
C_4_AŜH_12_	25 °C < T < 90 °C	0.893; 0.490; 0.447; 0.399; 0.365; 0.288; 0.278; 0.274; 0.245; 0.241; 0.236; 0.233
C_4_AŜH_10.5_	50 °C < T < 220 °C	0.816; 0.481; 0.471; 0.408; 0.375; 0.357; 0.287; 0.279; 0.271; 0.243; 0.234
C_4_AŜH_9_	90 °C < T < 370 °C	0.798; 0.492; 0.433; 0.398; 0.286; 0.280; 0.257; 0.248; 0.240
C_4_AŜH_6-x_	170 °C < T < 330 °C	0.616

Further investigation has demonstrated that no mineralogical changes arise between ~ 380 and ~ 700 °C. Meanwhile, at the end of the phase decomposition interval (at ~ 720 °C), the calcination of CaCO_3_ is seen, which lasts up to ~ 800 °C. Accordingly, the peaks of calcium oxide arise (*d* – 0.278 nm) (CaCO_3_ → CaO + CO_2_), which maximum intensities are seen at 940 °C. At the same time, above ~ 700 °C, the peaks of anhydrite (CaSO_4_) (*d* – 0.349 nm) are detected, indicating the beginning of the crystalline phase formation interval (700–1250 °C). These observations demonstrate that the first and the second temperature intervals slightly overlap. Additionally, the appearance of CaSO_4_ may mainly be attributed to the decomposition of calcium monosulfoaluminate, as shown in Eq. (1).

1 $${\text{C}}_{{4}} {\text{A}}\widehat{{\text{S}}}{\text{H}}_{{{12}}} \to {\text{CaO + Al}}_{{2}} {\text{O}}_{{\text{3(am)}}} {\text{ + CaSO}}_{{4}} {,}$$

2 $${\text{CaO + Al}}_{{2}} {\text{O}}_{{3}} \to {\text{CaAl}}_{{2}} {\text{O}}_{{4}} {,}$$

3 $${\text{CaSO}}_{{4}} {\text{ + 3CaO + 3Al}}_{{2}} {\text{O}}_{{3}} \to {\text{Ca}}_{{4}} \left( {{\text{AlO}}_{{2}} } \right)_{{6}} {\text{SO}}_{{4}} {,}$$

4 $${\text{CaSO}}_{{4}} {\text{ + 3CaAl}}_{{2}} {\text{O}}_{{4}} \to {\text{Ca}}_{{4}} \left( {{\text{AlO}}_{{2}} } \right)_{{6}} {\text{SO}}_{{4}} {,}$$

As the heating temperature rises to ~ 860 °C, diffraction peaks attributed to ye`elimite (C_4_A_3_Ŝ) (*d* – 0.375 nm) begin to appear, which gradually increase with the increasing heating temperature (up to 1250 °C). Simultaneously, peaks corresponding to the calcium aluminate phase (CA) (*d* – 0.297 nm) begin to appear at ~ 940 °C (Eq. (2)). However, with the temperature rise up to ~ 1100 °C, the diffraction maximums of CŜ, CA and CaO strongly attenuate, while those of ye`elimite are generated. This indicates that mentioned phases are consumed for the formation of ye`elimite through the solid-state reactions (Eqs. (3), (4)), and an increase in temperature promotes the interaction of the phases. It was observed that the critical temperature for CaSO_4_ and CA are at 1160 °C and 1200 °C, respectively. Exceeding these temperatures, the previously mentioned phases are no longer recorded in the XRD patterns. In the highest thermal treatment temperature (1250 °C), ye`elimite is observed as the dominating crystalline phase. Additionally, the small intensity peaks attributed to CaO also remain visible, showing incomplete solid-state reactions.

**Figure 8:**
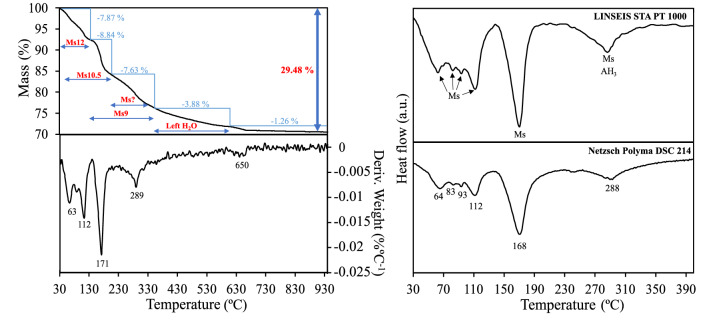
TG, DTG and DSC curves of the sample after hydrothermal synthesis.

In order to obtain more information about calcium monosulfoaluminate decomposition, simultaneous thermal analysis was performed. The thermal phase transitions that occurred during the sample heating are presented in Fig. 8.

According to obtained TG/DTG/DSC results, the thermal decomposition of the sample can be divided into 5 mass loss regions. The first region of the DSC curve reflects 4 endothermic peaks (at ~ 64 °C, ~ 82 °C, ~ 95 °C and ~ 112 °C), representing several chemical reactions that gradually took place. In comparison, only 3 effects incidents are seen in the DTG curve. Striving to ensure that the observed fourth peak in the curve is not an equipment noise, DSC measurements were carried out with another device (Netzsch Polyma DSC 214). Both devices confirmed the presence of a peak at ~ 95 °C in the DSC curves, which might reflect crystal structure changes of monosulfoaluminate since it is not observed in the DTG case. However, further research is needed to confirm it. Nevertheless, these effects can be assigned to the removal of adsorption water, the dehydration of the amorphous compound structure, and the release of the unbonded and loosely bonded water molecules in the interlayer of the calcium monosulfoaluminate structure. This observation corresponds well with the results of XRD in-situ analysis, which demonstrated the overlapping dehydration mechanism of different hydrate states of calcium monosulfoaluminate (Ms12 → Ms10.5 → Ms9) in this temperature interval.

The second temperature region has the highest mass loss (140–220 °C), and the effect at 168 °C corresponds to the continuation of the dehydration of the interlayer water (mainly transition of Ms10.5 → Ms9). Based on the XRD insights, it might be argued that dehydration of the main layers begins at the end of this temperature range. Meanwhile, the dehydration behaviour between 220 and 370 °C reflects not only the water loss from the main layers but also the remaining water loss out of the interlayers, i.e. dehydration of Ms9 and lower states. Additionally, at this temperature range, the Ms thermal effect overlaps with the amorphous AH_3_ thermal transition (230–300 °C)^33^. In the fourth region (370–600 °C), neither DSC nor DTG reflects any thermal transition. However, a gradual mass loss increase in the TG curve indicates the dehydration of remaining crystal water out of Ms main layers. In the last region, the thermal shoulder (~ 650 °C) corresponds to the decomposition of CaCO_3_.

### Insights of the work

**Figure 9:**
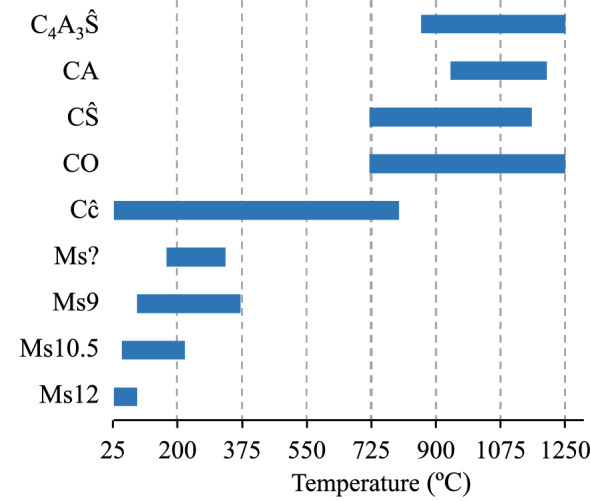
The ranges of crystalline phases developing in the thermal heating process of the synthesised sample detected by in-situ X-ray diffraction. Indexes: Ms – monosulfoaluminate (the number shows water content); Cĉ – calcium carbonate; CO – calcium oxide; A – anhydrite; CA – calcium aluminate; Y – ye`elimite.

The data of the current study complements the missing gaps in the thermal deterioration reaction sequences of calcium monosulfoaluminate and conversion into ye`elimite. The ranges of intermediate phases developing in the thermal heating process at 25–1250 °C are summarised and presented in Fig. 9.

As evident from the experimental data presented above, the dehydration of monosulfoaluminate occurs in a wide temperature range. The complexity of its structure (the presence of different amounts of water in the interlayers) causes the dehydration of four different hydration states of monosulfoaluminate (25–370 °C), which has been observed by in-situ X-ray diffraction. Additionally, the dehydration processes of different hydrate states of monosulfoaluminate do not take place sequentially (stepwise) but overlap. Accordingly, these results have also been confirmed by simultaneous thermal analysis: (1) the loss of mass is controlled by the removal of water from the interlayer structure and occurs in several steps, while (2) the observed water loss in the TG has poorly defined steps, that indicates the overlaps. Furthermore, this dehydration process distorted and disintegrated the crystalline structure of monosulfoaluminate, inducing the amorphization of the dehydration products. Therefore, this is the fundamental reason for limiting the application of X-ray diffraction in distinguishing the beginning and end temperatures of monosulfoaluminate main layers dehydration. Thus, further investigations are required to identify the exact dehydration temperature range of the main layers.

Meanwhile, the observation of solid-state reactions at elevated temperatures corresponds well with Y. El Khessaimi et al. examinations^34^, disclosing similar mechanisms of ye`elimite formation. In contrast, the onset of ye`elimite formation is observed at a lower temperature (860 °C) compared to the conventional ye`elimite synthesis method (1000 °C). Additionally, to the best of our knowledge, the in-situ X-ray diffraction approach to observe the rapid evolution of the microstructure during ye`elimite formation has not been previously published.

It should also be considered that results show the influence of drying conditions on crystalline cement hydrate at elevated temperatures. For instance, 50 °C can condition partial dehydration and shrinkage of monosulfoaluminate (Ms12 → Ms10.5 decrease ~ 9% in volume)^15^. In the long term, this can be a reason for concrete structures to deteriorate and adversely affect durability. Additionally, the temperature is essential for applying the drying techniques for sample preparation before characterising hydrated cement paste. Incorrectly chosen drying temperature can lead to the formation of different hydration states of monosulfoaluminate. Therefore, temperature and humidity must always be considered for adequate and comparable results.

Finally, it is important to underline that the identified stability ranges for different phases can be influenced and slightly altered by numerous changeable factors. Namely, grain size, degree of raw meal homogenisation, presence of impurities, experimental methods, etc. Therefore, it should be considered that the presented decomposition of monosulfoaluminate results might slightly differ in the conditions of real cementitious systems.

## Conclusions

The results of the present study demonstrate the reaction sequence by which calcium monosulfoaluminate 12-hydrate dehydrates at elevated temperature and, through solid-state reactions, converts into ye`elimite. The mechanochemical treatment (dry grinding at 900 rpm with 3 on–off cycles of 10 min) followed by the hydrothermal synthesis (for 8 h at 110 °C) was applied to produce monosulfoaluminate. The ye`elimite stoichiometry was used as a reference to prepare the initial mixture from pure raw materials (CaO, Al_2_O_3_, CaSO_4_·2H_2_O)_._

The X-ray diffraction results show that Ms14 is the dominant crystalline phase in the synthesis products immediately after hydrothermal treatment. However, after the drying process (50 °C 24 h), Ms14 loses two interlayer water molecules and transitions into Ms12. Consequently, QPA showed that the system consists of Ms12 (~ 54.8%), calcite (~ 1.9%), Ms10.5/Hc (~ 0.7%) and amorphous content (~ 42.6%). Additionally, it is determined that the amorphous content includes amorphous Al_2_O_3_ and AH_3_, as evidenced by FTIR, STA, SEM, ^27^Al NMR spectroscopy and mass balance calculation.

The thermal stability assessment revealed that the critical temperature for the dehydration of monosulfoaluminate is 25–370 °C. It was found that at this temperature, four different hydration states of monosulfoaluminate occur, with dehydration temperatures overlapping. Moreover, the data propose that the main interlayers’ dehydration of monosulfoaluminate might begin at ~ 200 °C. Within higher temperatures (720–1250 °C), solid-state reactions are observed. The results indicate that monosulfoaluminate recrystallised into CŜ, CA, CaO and C_4_A_3_Ŝ. Meanwhile, the further growth of ye`elimite at 1000–1250 °C results from the solid-state reactions between CŜ, CA and CaO. The presented results and insights enlarge knowledge of the thermal properties of monosulfoaluminate and could help to cement producers predict concrete’s behaviour at high temperatures.

## Data Availability

The datasets generated during and/or analysed during the current study are available from the corresponding author upon reasonable request.
